# Proposition for Extending the Requirements of a Collegiate Dental Education, so as to Embrace a Course of Lectures in a Medical School Proper

**Published:** 1853-01

**Authors:** B. Wood

**Affiliations:** Dentist, Nashville, Tenn.


### ARTICLE II.

*Proposition for Extending the Requirements of a Collegiate Dental Education, so as to embrace a Course of Lectures in a Medical School proper.* By B. WOOD, M. D., Dentist, Nashville, Tenn.

(Concluded from page 38.)

THE course of instruction in Dental Colleges being admitted to be inadequate to the wants of our profession, we come at once to inquire what are the means for its extension and improvement? Is it designed to increase the professorships in these institutions, thereby embracing a wider range of study? But does not the present number of professors and demonstrators occupy the full time of the collegiate hours, and if so, where is room for others unless by the exclusion of some of the existing chairs? Are some of these to give way for new ones? What department, then, is it proposed to extend, the medical, or the dental? Shall it be the former, and thus still further curtail the course in the latter which is already acknowledged to be "*barely sufficient* for giving instruction in practical and mechanical dentistry?" Or, in order to render this more thorough, shall the medical department be retrenched, thereby increasing the already too just cause for complaint on this score? Take which ever horn of the dilemma you will, and it necessarily narrows down a department already too limited.

The only way to make any substantial improvement, is to *extend the term of instruction.* And this, it is believed, ought to be done, by prolonging the time of study a year, and including an additional collegiate course. If a full and thorough training in operative and mechanical dentistry, could be had under competent preceptors, so as to allow the whole time of the colleges for the elucidation of theoretical principles, there would be less urgency for this; but since such tuition is not generally accessible, or, if it were, is not required by these institutions, three collegiate courses were certainly none too much to insist upon. Moreover, this would be no more than equivalent to the customary term of study in medicine, which, in the United States, is uniformly three years; in Europe it is at least one or two years longer, while at the same time the preliminary requirements are higher, the branches of study more diversified and the collegiate sessions longer. And, nearer home, we have an example, in the McGill College, Montreal, of a medical school requiring attendance upon *four courses of lectures of six months each.* The feeling throughout our own country is growing stronger and stronger in favor of a more prolonged term of medical study, and there can be little doubt of its being ere long brought about. Would it not be a triumphant thing for dental surgery to take the initiative in this important movement.

The extension of the term of dental study, so as to occupy three years and include three collegiate courses, could be done either by prolonging the term in dental colleges, or requiring a preliminary course in medicine. In favor of the latter there is this prominent reason, that it would afford a greater amount of medical information, and at the same time allow dental schools room for doing better justice to dentistry. It is necessarily the case with colleges that the same series of lectures is reiterated at each session, so that the *amount* of instruction would not be augmented by multiplying the courses in the dental schools; and two sessions, with proper attention and study, ought, perhaps, to suffice for impressing the principles inculcated. The importance also of taking a general survey of the cardinal medical branches, before the mind is directed to special details, is a consideration of moment. It appears there is an undue tendency on the part of students, to neglect the medical and theoretical branches taught in dental colleges. Professor Harris says, "the infirmary and mechanical rooms are so attractive that it requires all the talent of our colleagues, and the stringent regulations of the school, to obtain sufficient consideration for the medical branches;" which fact of itself sufficiently indicates the propriety of teaching such branches in a preliminary course, where the attention would not be thus divided; and it affords a strong motive for requiring previous attendance upon the lectures in medical schools proper. I shall now present, in a general way, some further considerations in favor of this mode of improvement.

The necessity for ampler facilities to acquire a knowledge of medicine than are afforded in dental colleges is urgent. Conscious of this we see their graduates not unfrequently resorting to a subsequent medical course, and it is believed there are very few who have not regretted the lack of means for this kind of instruction in dental colleges. That these are furnished with but two medical chairs, while some seven or eight are elsewhere found essential to do justice to the range of studies which go to constitute a medical education; and that these chairs are devoted to branches of a special rather than a general character, and are necessarily occupied to a great extent with minutia not taught in the ordinary schools of medicine, would seem to deprive the former of any just claim to the title of "medical" in the common acceptation of the term.

This is not said in depreciation of the ability and efficiency of the medical professors connected with dental colleges. Far from it. These gentlemen are no doubt fully competent to fill with credit any medical chair that might be assigned to them. Moreover in regard to those connected with the Baltimore college in particular, be it said that to them is due no ordinary credit for thus stepping forward in aid of the advancement of dental surgery while yet in its infancy. To them, indeed, is this branch of medicine much indebted for the views which have now become so general in regard to its true nature and station, a service which, when it shall have become fully accredited as a medical science cannot fail to claim its due reward of respect and gratitude from the entire medical profession.

But time and opportunity as well as talent are essential for the communication of this knowledge, and it will surely not be contended that two lecture hours a day during an ordinary collegiate term, be they occupied by whom they may, are adequate for imparting what it is incumbent upon the dental student to understand. Indeed, these chairs do not profess to inclued, however imperfectly, more than one-half of the *branches* included in the regular colleges. If there were a corps of some four or five teachers, so as to do *full* justice to the medical branches which are more directly important, there would be less cause for complaint for omitting the others. Still it were better that the student have the benefits of all, not one of which can be regarded as superfluous in his education, not one but is calculated to illumine his pathway in after practice.

A preliminary course in medicine, affording a full survey of all the medical branches, would, though it would be but for a single term, be the means of placing the dentist upon equal footing with the physician and surgeon. For the extension and application of the principles therein inculcated, to the specialty of dental surgery during the student's subsequent course, could not fail to refresh and fix them in his memory, so that even on the score of medical information he would not be far behind the graduate in medicine. The benefits of this, in mutual intercourse, in cases of consultation, in co-operation for the advancement of the common science, whether through individual or associate capacities, and in general concert of feeling and action, cannot be over estimated.

That such preliminary course must afford opportunity for more thorough instruction in dentistry during the term assigned for this, is too obvious to dwell upon. It would leave room not only for greater extension of the branches now taught but also for the introduction of new ones. A glance at the range of study in the dental colleges recently organized will suggest what additional branches might, with propriety and profit, be introduced.

The fact that new branches must arise as our science progresses, demanding a place in the curriculum of its colleges, affords a most cogent reason in favor of a previous course in medicine. The strong tendency of the dental mind for practical training to the exclusion of medical acquirements must eventuate in the gradual exclusion of the branches belonging to the latter, until nothing of medicine proper is left. And thus, sapped of its true foundation, dentistry as taught in dental colleges loses cast, becomes a sort of professionalized art, and, instead of being the ally, takes rank as the servant of medicine. Meanwhile with chairs of dental surgery in all the important medical colleges, (which such a state of things would necessitate,) this specialty, *as a science*, would come to be exercised by the regular profession. We might look to see medical dentists prescribing as a part of their *treatment* the service of the mechanical dentist; a state of things similar to what appears to exist already in Europe, judging from the frequency with which dental "surgeons" speak of sending their patients to their "ingenious mechanical friends" for the necessary artificial substitutes and appliances.

Such I believe will be the natural result of separate dental schools, without an extension of their present term of study. At first the medical *professors* will be excluded for practical dentists, who, though perhaps graduates in medicine, have, from special attention to a single branch, lost sight to the importance of general principles. Then the medical *branches* will be gotten rid of; and, as the clamor increases for practical and mechanical training, the theoretical branches will give way, and these institutions degenerate to workshops. If this were guarded against so as to maintain a medical character, the evils could scarcely be less; mechanical dentistry would be forced to the goldsmiths for want of attention, and it would be apt to carry operative dentistry with it; leaving nothing worth cultivating which could not be equally cultivated in medical colleges.

Upon the other hand, suppose the course of instruction be extended, while yet dental surgery is kept isolated and disconnected from the general science; this *separation* could hardly fail to induce greater and still greater discrepancy in the mode of instruction of each; and this, as has been suggested, were but the prelude to separate modes of medical practice, resulting in a system of dentalized medicine; a sort of "new school" of practice, in hostile antagonism to the old system, and ending in discord and animosity alike detrimental to both.

That such would be the tendency is obvious from the nature of things and the history of medical science. Exclusive attention to specialties lead to partial views, and this it is that originates partial and exclusive doctrines. Exclusive attention to the virtues of a few plants gave rise to botanic medicine, based upon lobelia and pepper; hydropathy arose from the like attention to the good effects of water in certain cases; so also a system of diagnosis and practice has come from the inspection of a single excretion. Now, if things so trivial suffice to originate new theories and afford bases for rival modes of practice, how much more should a congeries of organs, like those of the mouth, and a class of remedial agents, like those employed in dental surgery? The mouth and tongue have always been important points of diagnosis. The teeth themselves afford indications of constitutional conditions. Here the dentist has peculiar advantages for observation; and upon this basis might be erected a rival system of medicine as specious as it would be formidable. If then the *means* exist, who can doubt that, if favored by the *conditions*, they will be brought into excercise? And what more favorable than the creation of separate and exclusive schools of medicine for the cultivation of so important a specialty. Alienated from the general science, and cultivated with an exclusive spirit, and yet as a medical science, dental surgery can hardly fail to give origin to a new system of medical practice. Confined to this specialty from the first, isolated from students designing themselves for general practice, and educated by a dissimilar mode of instruction, and under teachers devoted to a distinct profession, the dentist could have but little concert of action, or sympathy of thought and feeling with the regular physician. Medicine, as represented by the two, would be no longer a unit. It becomes "a house divided against itself." Adverse doctrines and rival modes of practice begin to prevail in each division. The younger profession rises up in defiant attitude against the older, and the older scoffs at and derides the younger. And then it needs but some master spirit to lead in order to make the breach impassable and the warfare perpetual.

If, on the contrary, dentists are required to become well grounded in medicine, at the regular colleges, a union between the two professions is at once effected. The dismembered parts of a common science are connected by a natural link, lending mutual support to each other and giving unity and strength to the whole.

But apart from the general considerations in favor of this, it would not be inconsistent with the plan of organization of dental colleges, nor, do I believe, prejudicial to them pecuniarily, but rather to their interests. A course in a medical school is already accounted equivalent to one in those institutions, showing that this is regarded as a legitimate part of dental instruction, and if *needed* why should it not be *required?*

It has been recommended by the American Medical Association to examine first-course students, and give them certificates of proficiency, &c. Such certificate, (which could be regarded as an honor equal to that of Bachelor of Medicine, formerly granted,) and a full course in a dental school, being prerequisite to graduation in the latter, would give a title to the degree of D. D. S., beyond dispute, which being thus made more honorable would be more sought after. Opposition to dental schools would then cease, and their claims as schools of medicine could be advocated with a better grace. It is vain to attempt to conceal or shuffle over the great and valid objection to these institutions, arising from the inadequacy of their range of instruction considered as a warrant for a doctorate, an objection which has ever been felt, and which must eventuate in their overthrow unless means be adopted for its removal.

If dental colleges had been brought in operation as *supplementary* to medical schools, they had probably succeeded better. For the statistics show that it is by the patronage of *medical men,* (including practicing dentists, whose gĕneral information must be supposed to have been respectable,) that they have been mainly supported. In the American Journal of Dental Science, (1851,) a writer evidently advised in regard to the facts, says: "at the present time the number of graduates from this institution (the Baltimore College) is about one hundred—of these, nineteen had previously earned the degree of doctor of medicine," (vol. 2, page 69.) "Of the graduates of the Ohio Dental College, *over one half* had practiced from four to twenty years, at the time of entering the institution. The same can probably be said of the college in Baltimore." (*Ibid.* p. 70.) If to these were added those who had taken a single course in medicine, or had availed themselves of other means for obtaining medical information, it would doubtlessly show that by far the greater portion, and perhaps nearly all, have taken the dental course as a supplementary part of their education.

Now if medical men have been the principal patrons of dental schools, would there be any great danger in looking exclusively to them for support? and could it be considered over-exacting to require of the tyro an acquaintance with medicine before admission to their privileges and honors? And, if a knowledge of medicine is needed, would it not be better for the student, to require it at the start, than that, having subsequently found the need of it, he should feel constrained to seek it at the medical colleges? Let the need of such knowledge be emphatically proclaimed on the part of dental schools, by the nature of their requirements, and there is no doubt the "conditions of graduation" would be generally submitted to.

In the foregoing pages I have not thought it necessary to urge the importance of special schools of dental surgery. Nor have I spoken of the claims of, or the advantages to be derived from, those which are now in operation; the *object* of this communication being to point out deficiencies and imperfections in their system of instruction, considered as a means of a full collegiate education; thereby illustrating the need of the proposed improvement and the obligations which exist in favor of its adoption. But, not to be misunderstood, I would here say, distinctly, that it is not the *management*, but the *system* of these institutions that is herein objected to. They are certainly not wanting in talent and zeal sufficient to claim patronage. If conducted with sufficient ability to be useful as a means of instruction to the physician and well informed dental practitioner, the tyro ought surely to be satisfied on this score. And whatever may be said of their *tendency* upon the ultimate condition of our profession they are unquestionably valuable, as supplying, to a considerable extent, its present wants. In absence of other instruction they should by all means be resorted to, and notwithstanding the very best private and medical tuition were had, they would no doubt well repay the time and expense of attending them. And although less adequate to the full wants of the dentist than assumed, yet, being the only means of dental instruction that has been generally accessible, the fact of attending them, should of itself be something of a guarantee to confidence, as evincing an active disposition to secure every means of improvement, which very disposition is, in truth, an almost unerring precursor of final success. The advantage also of some distinctive title like that which they confer, especially to the young dental practitioner, unsustained by an acquired reputation, is freely conceded.

These considerations, indeed, afford a strong restraint to the public expression of one's honest objections; lest empirics may therein find a warrant for assuming the calling of the dentist without any adequate instruction or guarantee of qualification. But the restraint here seemingly imposed is specious, not valid, and if effective must result in the very evil which it fain would avoid. For, if the course in dental schools is held up as complete in itself for instruction in the whole range of dental surgery, why should not the amount of training which any quack can lay claim to, be warrant enough for his assuming proficiency in some of the minor and "merely mechanical" branches? Thus reason the masses. Let it, however, be known and avowed that even these institutions are inadequate for the demands of our art, and the quack is at once discarded. For who will countenance ignorance in a vocation requiring so high a grade of qualification?

But the justification for a free discussion of this matter looks beyond the present and temporary effect upon empiricism, whether for good or ill. The motive object is the perfection of our science and the advancement of our profession—and, incidentally, the separation of the educated dentist from the charlatan by a broader line of demarkation.

We could not hide the imperfections of the present system of dental instruction if it were desirable to do so. It has been the continued complaint that the "better educated class of dentists stand aloof." Is this *all* in consequence of selfishness, &c.? Why then do not the medical profession recognize dental graduates as belonging to the fraternity? Will a course in dentistry be received at their colleges as equivalent to a course in medicine? Have not medical associations seen fit to reject graduates of dental schools as unqualified for fellowship? These are disagreeable facts it is true, but it is no use to give them the go-by; it is worse than useless to carp at them. Let them be looked steadily in the face; let the *true cause* be unravelled, and let *this* be removed.

These remarks are not made by way of disparagement to the medical attainments, much less the operative proficiency of graduates of dental surgery in general; many no doubt have attained the highest grade of qualification; but the same may be said of many who can boast of no title whatever. And the result, in both cases, is probably due to the same cause, viz. a persevering aspiration, which stops not at the limits of any system of tuition, but relying upon its own strength and guidance, presses still onwards. Neither do I say that the *mere* graduate in dentistry, may not have a claim to consideration and respect as a medical man. All that I assume is, that his course of study does not qualify for taking rank, at once, in the medical profession, "side by side with the physician and surgeon;" while I contend that it ought to be such as to enable him to do so.

If the positions assumed in the foregoing remarks are correct and the main deductions legitimate, if a course of medical instruction is needed as a part of a collegiate dental education, and no serious obstacle exists to forbid the extension of the scheme of dental colleges so as to include such a course, there would certainly seem to be the strongest inducements to carry it out in practice. Nearly every argument that has been urged in support either of dental schools as now organized, or of dental lectureships as proposed, is equally applicable in favor of this.

The influence of these schools upon the cause of dental education, should, of itself, furnish them with the strongest motive for its adoption. If, instead of lending the weight of their example against the necessity of thorough medical acquirements, by separating themselves from the institutions devoted to the general science, and asking no adequate preliminary qualification in the latter, they were decidedly and unequivocally to declare in favor of such qualification, by requiring as an indispensable condition to graduation a course of instruction in medicine, it would at once impress the community with a due sense of the nature, dignity and prerogatives of our profession; the good effects of which, in promoting the general interests of our science and the prosperity of its institutions, would be incalculable.

